# The Center for Epidemiologic Studies-Depression (CES-D) scale measures a continuum from well-being to depression: Testing two key predictions of positive clinical psychology

**DOI:** 10.1016/j.jad.2017.02.015

**Published:** 2017-04-15

**Authors:** Andy P. Siddaway, Alex M. Wood, Peter J. Taylor

**Affiliations:** a Behavioural Science Centre, Stirling Management School, University of Stirling, Stirling, Scotland, UK; b Division of Psychology & Mental Health, University of Manchester, Manchester, England, UK

**Keywords:** Recovery, Stigma, Therapy, Intervention, Prevention, Well-being

## Abstract

**Background:**

Two core but untested predictions of Positive Clinical Psychology (PCP) are that (1) Many psychiatric problems can be understood as one end of bipolar continua with well-being, and (2) that reducing psychiatric symptoms will provide an equal (near linear) decrease in risk for several other psychiatric variables, irrespective of position on continua.

**Aims:**

We test these predictions in relation to a purported well-being/depression continuum, as measured by the Center for Epidemiologic Studies-Depression (CES-D), a popular measure of depressive experiences in research and clinical practice.

**Method:**

A large (*N*=4138), diverse sample completed the CES-D, which contains a mixture of negatively worded and positively worded items (e.g., “I felt sad,” “I enjoyed life”). The latter are conventionally reverse scored to compute a total score. We first examined whether purportedly separate well-being and depression CES-D factors can be reconceptualised as a bipolar well-being/depression continuum. We then characterised the (linear or nonlinear) form of the relationship between this continuum and other psychiatric variables.

**Results:**

Both predictions were supported. When controlling for shared method bias amongst positively worded items, a single factor well-being/depression continuum underlies the CES-D. Baseline levels on this continuum are found to have near linear relationships with changes in anxiety symptoms, aggression, and substance misuse over time, demonstrating that moving from depression to well-being on the CES-D provides an equal decrease in risk for several other psychological problems irrespective of position on the continuum.

**Limitations:**

The CES-D does not measure well-being as comprehensively as established scales of well-being.

**Conclusions:**

Results support calls for mental health services to jointly focus on increasing well-being and reducing distress, and point to the value of early intervention and instilling resilience in order to prevent people moving away from high levels of well-being.

## Highlights

• Two key predictions of Positive Clinical Psychology (PCP) are supported.
• The Center for Epidemiologic Studies-Depression (CES-D) measures a depression/well-being continuum.
• The continuum has near linear relationships with other psychiatric variables over time.
• Results support calls for mental health services to jointly focus on increasing well-being and reducing distress.

Well-being is becoming an increasingly central focus of international policy (e.g., Department of Health, 2009; Mental Health Commission of Canada, 2009). The positive psychology movement was proposed in order to raise awareness of the importance of researching psychological traits and constructs that promote well-being (Gable and Haidt, 2005, Seligman and Csikszentmihalyi, 2000). This literature has burgeoned and recent years have seen an increasing shift towards a broader position that jointly focuses on alleviating psychological problems *and* promoting well-being (e.g., Ivtzan et al., 2015; Joseph and Wood, 2010; McNulty and Fincham, 2012; Peterson, 2006; Wood and Tarrier, 2010), which some have labeled the “second wave” of positive psychology (Ivtzan et al., 2015, Wong, 2011).

One prominent component of this shifting zeitgeist has been the inception of Positive Clinical Psychology (PCP), which has called for positive psychology research to be integrated with the voluminous evidence base concerned with understanding and treating psychological problems (Wood and Tarrier, 2010, as clarified in Johnson and Wood, in press). Numerous articles (e.g., Johnson and Wood, in press; Lomas, 2015; Joseph and Wood, 2010; Wood and Tarrier, 2010; Wong, 2011) and at least three books (Ivtzan et al., 2015, Peterson, 2006, Wood and Johnson, 2016) have now summarized evidence which supports this integration. These demonstrate, for example, that: (i) constructs studied by positive psychology researchers can independently predict psychological problems over and above clinical constructs; (ii) that constructs studied in positive psychology can confer resilience to psychological problems; and (iii) that interventions which aim to move people towards well-being can also be used to help people move away from psychological problems, either in isolation, or alongside clinical interventions.

Although this progress is promising, the notion that positive psychology research can and should be integrated with the existing evidence base for psychological problems remains largely untested. This study explores this issue by testing two core predictions made by PCP using the example of depressive experiences. The first prediction to be tested here is the idea that many psychological problems in fact form continua with well-being. A well-being/psychological problem continuum would indicate that research on either area has implications for the opposite pole (Joseph and Wood, 2010, Wood and Tarrier, 2010) and would suggest that the language of “positive” and “negative” is arbitrary and dependent on one's perspective and context (Johnson and Wood, in press; McNulty and Fincham, 2012; Wood and Johnson, 2016). This conceptualization would align with the substantial research base demonstrating that psychological problems are best-viewed as continuous constructs rather than discrete categories (e.g., see Bentall, 2003; Haslam et al., 2012; Markon et al., 2011) and which has examined continuously distributed transdiagnostic constructs and mechanisms (e.g., see Harvey et al., 2004).

We test this key prediction in relation to a hypothesized depression/well-being continuum using the Center for Epidemiologic Studies-Depression (CES-D) scale (Radloff, 1977). The CES-D is one of the most frequently used self-report measures of depressive experiences (Santor et al., 2006) and there is extensive support for its psychometric properties (Ensel, 1986, Radloff, 1977, Roberts, 1980, Shean and Baldwin, 2008). The fact that the CES-D contains a mixture of negatively worded items (e.g., “I felt sad;” “I thought my life had been a failure”) and positively worded items (e.g., “I felt happy;” “I enjoyed life”) led to the proposal that it could be re-conceptualized as a depression/well-being continuum (Joseph, 2006, Joseph, 2007). It was argued that for a score of zero to occur on the CES-D, a person would have to give all of the negatively worded items (e.g., “I felt sad”) the lowest possible score (“rarely of none of the time”) *and* all of the positively worded items (e.g., “I enjoyed life”) the highest possible score (“most or all of the time”). For such a person it would be misleading to state that they have merely indicated an absence of depressive experiences; such an individual has also clearly indicated the *presence* of well-being (Joseph, 2006, Joseph, 2007, Joseph and Wood, 2010).

One existing study has tested whether the CES-D can be re-conceptualized as a well-being/depression continuum (Wood et al., 2010). The authors found that when accounting for item wording using structural equation modeling, the CES-D can indeed be understood as measuring a bipolar continuum that ranges from well-being to depression. The authors established these findings using separate adult and older adult samples and presented evidence that the well-being items (e.g., “I felt happy;” “I enjoyed life”) demonstrate convergent validity with the well-validated Scales of Psychological Well-being (Ryff, 1989). Given the potential practical importance of the suggestion that depression forms a bipolar continuum with well-being, and the increasing emphasis on replicating scientific findings to ensure that they are robust and generalizable, our first aim was to replicate the structural analyses of this previous study using a large, diverse sample of adolescents.

The second prediction made by PCP that we test here is the idea that moving along the well-being/depression continuum (total score on the CES-D) towards well-being will provide an equal decrease in risk for several other psychological problems, irrespective of position on the continuum (Wood & Johnson, in press; Wood and Tarrier, 2010). We test this prediction by examining the form of the relationship between the depression/well-being continuum and other psychological problems over time, which Flett et al. (1997) referred to as “phenomenological continuity.” That is, continuity in the relationship between psychological problems and their antecedents, concomitants, or sequalae. Accordingly, even if depression is relatively continuous in a psychometric sense, its relationship with associated variables could be relatively discontinuous or nonlinear in form, defining a natural boundary of depressive experiences (Markon, 2010).

The benefit of these analyses is that they make the clinical importance of this topic more apparent and explicit than merely examining whether a well-being/depression continuum exists. One possibility, for example, is that there is no relationship between the well-being/depression continuum and other psychological problems up to a particular point (e.g., throughout the range of the well-being pole), after which the detrimental consequences of depression begin to manifest. Evidence of this relationship would corroborate the current emphasis in mental health services on alleviating and treating psychological problems. This conceptualization of depression (and other mental health) problems underpins psychiatric nomenclature and, as a result, psychiatric and psychological interventions tend to be stopped at the point of problem absence.

An alternative possibility is a linear relationship between the well-being/depression continuum and other psychological problems throughout the range of the continuum. This relationship would be apparent if depressive experiences increase at a constant rate along with other psychological problems, without any threshold defining a change in association. Evidence of this relationship would simultaneously highlight the importance of treating depression (because as depressive experiences increase, so do other psychological problems) and emphasize the usefulness of fostering well-being (because as well-being increases, psychological problems decrease). Such evidence would be consistent with calls from professional bodies (e.g., The British Psychological Society, 2010) and the mental health recovery movement (e.g.,Andresen et al., 2003) for mental health services to focus not just on tackling psychological problems but also on fostering well-being and helping people live a valued, meaningful life.

We examine these two key predictions of PCP using a large population-based archival dataset, which, by implication, involved variability in the latent entity, thereby minimizing the likelihood of systematic sampling bias, which could have been introduced had we used a purely community or clinical sample (Waller and Meehl, 1998). For example, using an undergraduate sample could introduce a systematic sampling bias since only those individuals functioning well enough to attend classes would be studied (Hankin et al., 2005). Likewise, focusing entirely on clinical individuals may limit variability in depressive experiences (Hankin et al., 2005) as clinical samples often exhibit more severe symptoms and greater comorbidities than population-based samples (Newman et al., 1998).

## 1 Method

### 1.1 Participants

The sample comprised 4138 adolescents and adults aged 13–21 years from Hawai‘i. These individuals took part in the five-year longitudinal Hawaiian High Schools Health Survey (HHSHS) study conducted by the National Center on Indigenous Hawaiian Behavioral Health (NCIHBH). This sample provides a broad spread of ages, ethnicities, socioeconomic status,’ and gender (Andrade et al., 2006, Hishinuma et al., 2000). Participants for the HHSHS study were sampled from five high schools which were selected from both urban and rural areas to obtain a representative sample of adolescents residing in Hawaii. Students who provided assent completed the survey in their classrooms under the supervision of their teachers. Parents of students younger than 18 years old were notified of the study by mail and given an opportunity to refuse participation. Data collected during the 1992/1993 (*N*=4164), 1993/1994 (*N*=4182), and 1994/1995 (*N*=1433) school years were used in this study. There was some missing demographic information and incomplete questionnaire responses (see Andrade et al., 2006; Hishinuma et al., 2001) and we multiply imputed missing data as best practice (discussed below).

### 1.2 Measures

#### 1.2.1 Center for Epidemiological Studies-Depression (CES-D; Radloff, 1977)

CES-D responses capture the frequency of feelings and behaviours over the past seven days and are rated on a 4-point scale ranging from 0 (rarely or none of the time) to 3 (most or all of the time). The CES-D contains 20 items that are summed so that scores have a potential range from 0 to 60, with higher scores indicating greater frequency of depressive experiences (Radloff, 1977). Numerous studies have examined the factor structure of the CES-D (McArdle et al., 2001, Prescott et al., 1998, Radloff, 1977) and these have generally suggested that negative and positive items load onto separate factors (see Shafer, 2006, for a review). There is extensive support for the psychometric properties of the CES-D, including high internal consistency in community and clinical populations (Cronbach's αs .85−.90; Ensel, 1986; Radloff, 1977; Roberts, 1980); convergent validity with other popular measures of depressive experiences such as the Patient Health Questionnaire-9 (r=.85; Amtmann et al., 2014) and Beck Depression Inventory-II (Shean and Baldwin, 2008); and divergent validity from, for example, aggression (r=.44) and substance use (r=.24; Makini et al., 1996). A cutoff score of 16 has been found to have sensitivity and specificity rates of 86.7 and 76.6 for identifying depressed individuals, whereas a cutoff score of 21 has a sensitivity and specificity rate of 73.0 and 96.1 (Shean and Baldwin, 2008). The CES-D demonstrated excellent internal consistency in the current sample (α=.88).

#### 1.2.2 State-Trait Anxiety Inventory-Trait scale (STAI; Spielberger et al., 1970: Trait subscale)

Trait anxiety is seen as a relatively stable individual difference in the tendency to respond to situations perceived as threatening with elevation in state anxiety (Spielberger et al., 1970). Items are rated on a 4-point frequency scale based on “how you generally feel.” Support for the psychometric properties of the STAI has been extensive (see Spielberger, 1989; Spielberger et al., 1970). The trait scale demonstrates excellent internal consistency (average Cronbach's αs>.89); excellent test–retest reliability at multiple time intervals (average *r*=.88; Barnes et al., 2002); adequate convergent validity with other measures of anxiety (Spielberger, 1989); and divergent validity from, for example, aggression (*r*=.38) and substance use (*r*=.19; Knight et al., 1983). The current sample demonstrated excellent internal consistency (α=89).

#### 1.2.3 Substance Abuse Subtle Screening Inventory—Adolescent version (SASSI-A; Miller, 1990)

The SASSI-A is a brief screen for substance use, impairment, and dependency arising from substance use. The SASSI-A has been shown to have good psychometric properties, including acceptable internal consistency in the current sample (α=.74); and divergent validity from anxiety (*r*=.19), depression (*r*=.24), and aggression (*r* =.33; Makini et al., 1996). It has also been shown to concord with a diagnosis of substance abuse and dependency on the Diagnostic Interview Schedule for Children (Nishimura et al., 2001), predict counselor DSM-III diagnoses for dually diagnosed adolescent inpatients (Piazza, 1996), and predict adolescent chemical dependency (Risberg et al., 1995).

#### 1.2.4 Braver Aggressiveness Dimension Scale (BADS; Braver et al., 1986)

The BADS is a 14-item abbreviated self-report measure of child and adolescent aggression. It was derived from the longer Youth Self-Report scales (YSR: Achenbach, 1991), the self-report version of the Child Behaviour Checklist. Items selected for the BADS were those items from the YSR which were significantly more likely to be endorsed by clinically-diagnosed, conduct disordered children and adolescents. The BADS has good psychometric properties, including good internal consistency in the current sample (α=.85), one year test–retest stability (*r*=.61), and divergent validity (Makini et al., 1996).

### 1.3 Missing data

There were substantial amounts of missing data 6.4% of all values were missing for the 1992/1993 school year, 52.2% of all values were missing for the 1993/1994 school year, and 22.53% of all values were missing for the 1994/1995 school year. The missingness was not completely at random (MCAR). We addressed this potential problem by multiply imputing missing data on all variables at the item level using SPSS version 21.0 (IBM Corp, 2012). Multiple imputation (MI) is increasingly advocated as the optimal approach for dealing with missing data (Graham, 2009, Schafer and Graham, 2002, Shrive et al., 2006). When MI has been compared with alternative methods of handling incomplete data (e.g., single imputation methods, complete-case analyses, maximum likelihood approaches), it has been shown to generate less biased estimates that have more statistical efficiency (e.g., Crawford et al., 1995; Donders et al., 2006; Liu and Gould, 2002; Tang et al., 2005). There is also evidence indicating that MI performs well across different circumstances, such as small samples, very large multiple regressions, and when there are large amounts of missing data (Graham and Schafer, 1999).

MI works by generating plausible missing values multiple times based on the distribution of the observed data. Random components are incorporated into these estimated values to reflect their uncertainty. This procedure creates a set of ‘‘complete’’ data sets with no missing values. Analyses are then run separately on each data set, and the results are pooled across datasets using multiple imputation combining rules (Enders, 2010, Graham, 2009). The purpose of MI is not to obtain the individual values themselves but to estimate unbiased parameter estimates of the data set as a whole (Graham, 2009). We followed recommendations to match the number of imputations to the fraction of missing information because progressively larger numbers of imputed datasets are needed to maximize power in subsequent significance testing (Bodner, 2008, Graham et al., 2007, White et al., 2011).

### 1.4 Statistical analysis

Analyses were conducted using SPSS version 21.0 (IBM Corp, 2012) and R (R Development Core Team, 2009). The CFAs were performed using the R lavaan package, version .5–18 (Rosseel, 2012). Complete cases were used for these analyses and three CFA models were tested. Model 1 was the standard two factor model consisting of separate negatively worded (e.g., “I felt sad”) and positively worded (e.g., “I enjoyed life”) items, which were allowed to correlate. Model 2 was a single factor model with all items loading on a single factor. Model 3 also featured a single substantive well-being/depression factor, however the positively worded items were allowed to cross-load onto a second methodological artefact factor which accounted for additional residual inter-correlation between these items.

As CES-D data are ordinal, we employed WLSMV estimation (Flora and Curran, 2004). Acceptable fit was operationalized as Root Mean Squared Error of Approximation (RMSEA)<.08, Comparative Fit Index (CFI)>.90, and Tucker Lewis Index (TLI)>.90. Good fit was operationalized as RMSEA<.06, CFI>.95, and TLI>.95 (Hu and Bentler, 1999). The fit of competing CFA models were compared using: (i) Akaike's Information Criterion (AIC; calculated using Maximum Likelihood CFA), which tests the relative fit of competing models after adjusting for parsimony (lower AICs indicate less information loss and thus a superior model), and (ii) CFI, using a .002 cutoff (Meade et al., 2008). Both approaches are argued to be superior to using the chi-square statistic to compare model fit because this statistic is known to be highly sensitive to sample size (see Meade et al., 2008).

Hierarchical ordinary least squares (OLS) regressions were used to explore linear and nonlinear relationships between CES-D scores, treated as a single factor (all items summed to produce a total score), and outcome variables. Regressions were conducted using MI data. In each analysis, Step 1 involved fitting a model whereby CES-D total scores had a linear relationship with the outcome variable measured at the same time (1992/1993 school year), or measured at follow-up 1 or 2 years later (1993/1994, 1994/1995 school years), whilst controlling for scores on the outcome variable at baseline (hence it was the change in outcome that we were predicting). Steps 2 and 3 tested whether adding a nonlinear term (squared and cubed CES-D total scores) made a significant improvement to the amount of variance explained. Improvement in model fit was based on Δ*R*^*2*^. Statistically significant deviations from linearity were graphed in order to visually display relationships, using unstandardized regression coefficients. This also clarified whether nonlinearity was substantive.

### 1.5 Comparison of CFA models

Table 1 shows that the two factor model (Model 1) demonstrated an improvement in fit over the one factor model (Model 2), replicating previous findings regarding the factor structure of the CES-D (see Shafer, 2006). Model 3 (a well-being/depression continuum) demonstrated an improvement in fit again, thereby replicating the findings of Wood et al. (2010). The AIC statistic, which accounts for model complexity, and the change in model fit according to CFI (Meade et al., 2008), both pointed to the superiority of Model 3, as hypothesized. These results indicate that when shared method bias amongst positively worded items is controlled for, a single factor underlies the CES-D items. Furthermore, as we argued in the Introduction, Model 3 was also favored on theoretical grounds because endorsing positively worded items, which are usually reverse-scored (e.g., “I felt happy,” “I enjoyed life”), does not merely indicate the absence of depressive experiences; it actually indicates the *presence* of well-being (Joseph, 2006, Joseph, 2007, Joseph and Wood, 2010).

**Table 1:** Comparison of three mean- and variance-adjusted weighted least squares CFA models.

	Model fit					
Model	*Χ*^*2*^	*df*	AIC	TLI	CFI	RMSEA
1. Two factor	4209.891*	169	163260.390	.920	.929	.082
2. Single factor	8025.236*	170	165187.856	.845	.861	.115
3. Single factor, method variance factor	3921.218*	166	163175.121	.924	.934	.080

*Note. N*=4138; CES-D completed during 1993/1994 school year; analyses are reported to three decimal places for clarity; AIC statistic produced from Maximum Likelihood CFA.

* *p*<.001.

### 1.6 Exploration of linear and nonlinear relationships with outcome variables

**Fig. 1:**
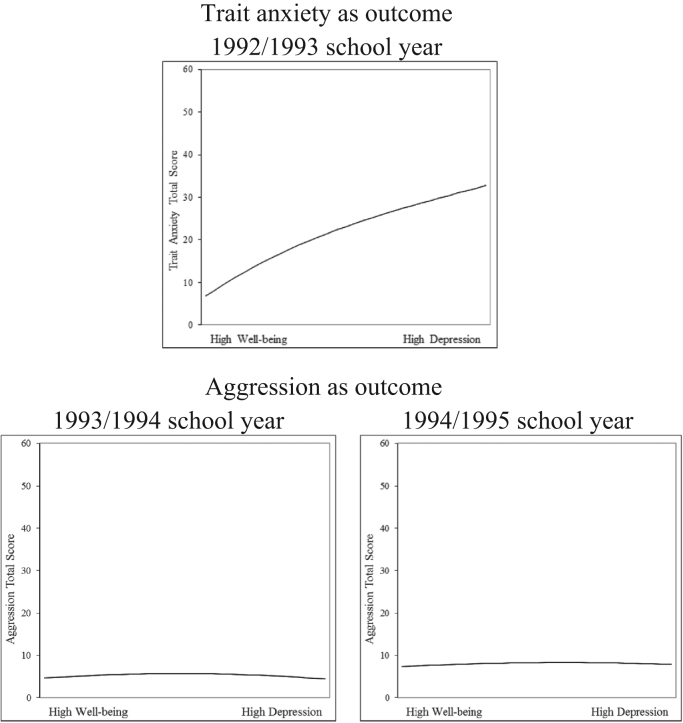
Line graphs plotting unstandardized nonlinear regression lines for statistically significant Δ*R*^*2*^ values. Total CES-D scores predict outcome variables at different time points; CES-D scale completed during 1992/1993 school year.

A series of regression analyses were conducted to explore the form of the relationship between the CES-D and outcome variables over time (Table 2). Three of the eight regression models showed statistically significant nonlinear relationships for Step 2. However, the squared term accounted for very little additional variation above and beyond the linear main effect (.7%, .1%, .2%). One of the eight regression models showed statistically significant nonlinear relationships for Step 3. However, again, the cubed term accounted for very little additional variation (.6%). Thus, in all cases the nonlinear term failed to make any substantive improvement to the original linear model and results provide only very weak evidence of a nonlinear relationship. The observed nonlinearity appears to be of statistical but not clinical significance and the relationship between the well-being/depression continuum and outcome variables appears to be near linear over time. Statistically significant nonlinear relationships were also explored graphically (Fig. 1). The graphs reveal only subtle variation away from perfect linearity.

**Table 2:** Results of regression analyses comparing linear and nonlinear effects of CES-D upon change in outcome.

Step	Outcome variables		*B*	*SE B*	*β*	Δ*R*^*2*^
		Trait anxiety				

1992/1993 school year (*N*=4069)						
1	Constant		11.243	.194		
	Total CES-D score		.702	.010	.744***	.554***

2	Constant		9.347	.310		
	Total CES-D score		.952	.034	1.008***	
	Total CES-D score squared		−.006	.001	−.277***	.007***

3	Constant		6.815	.446		
	Total CES-D score		1.493	.077	1.581***	
	Total CES-D score squared		−.034	.004	−1.615***	
	Total CES-D score cubed		.000	.000	.813***	.006***
		Aggression				

1992/1993 school year (*N*=4069)						
1	Constant		1.784	.124		
	Total CES-D score		.249	.006	.523***	.274***

2	Constant		1.969	.199		
	Total CES-D score		.225	.022	.472***	
	Total CES-D score squared		.001	.000	.054	.000

3	Constant		1.695	.292		
	Total CES-D score		.283	.051	.595***	
	Total CES-D score squared		−.002	.002	−.234	
	Total CES-D score cubed		.000	.000	.175	.000

1993/1994 school year (*N*=4101)						
1	Constant		5.334	.145		
	1992/1993 BADS total score		.354	.018	.380***	
	Total CES-D score		.027	.008	.062***	.176***

2	Constant		4.994	.224		
	1992/1993 BADS total score		.354	.018	.381***	
	Total CES-D score		.072	.024	.162*	
	Total CES-D score squared		−.001	.001	−.105*	.001*

3	Constant		4.660	.314		
	1992/1993 BADS total score		.354	.018	.380***	
	Total CES-D score		.144	.053	.324^**^	
	Total CES-D score squared		−.005	.003	−.482	
	Total CES-D score cubed		.000	.000	.228	.001

1994/1995 school year (*N*=4101)						
1	Constant		7.897	.211		
	1992/1993 BADS total score		.244	.023	.302***	
	Total CES-D score		.020	.009	.051*	.115***

2	Constant		7.573	.254		
	1992/1993 BADS total score		.244	.023	.303***	
	Total CES-D score		.062	.023	.161*	
	Total CES-D score squared		−.001	.000	−.116*	.002*

3	Constant		7.366	.331		
	1992/1993 BADS total score		.244	.023	.302***	
	Total CES-D score		.106	.052	.277*	
	Total CES-D score squared		−.003	.002	−.384	
	Total CES-D score cubed		.000	.000	.163	.001
		Substance misuse				

1992/1993 school year (*N*=4069)						
1	Constant		.262	.038		
	Total CES-D score		.045	.002	.336***	.113***

2	Constant		.259	.062		
	Total CES-D score		.045	.007	.339***	
	Total CES-D score squared		.000	.000	−.003	.000

3	Constant		.284	.090		
	Total CES-D score		.040	.016	.299*	
	Total CES-D score squared		.000	.001	.253	
	Total CES-D score cubed		.000	.000	.151	.000

1993/1994 school year (*N*=4101)						
1	Constant		1.122	.041		
	1992/1993 SASSI-A total score		.397	.016	.436***	
	Total CES-D score		.006	.002	.050*	.208***

2	Constant		1.059	.059		
	1992/1993 SASSI-A total score		.397	.016	.436***	
	Total CES-D score		.014	.006	.119*	
	Total CES-D score squared		.000	.000	−.071	.001

3	Constant		1.009	.086		
	1992/1993 SASSI-A total score		.397	.016	.436***	
	Total CES-D score		.025	.015	.207	
	Total CES-D score squared		−.001	.001	−.277	
	Total CES-D score cubed		.000	.000	.125	.000

1994/1995 school year (*N*=4101)						
1	Constant		1.754	.056		
	1992/1993 SASSI-A total score		.221	.020	.297***	
	Total CES-D score		.005	.002	.052*	.098***

2	Constant		1.681	.071		
	1992/1993 SASSI-A total score		.220	.020	.296***	
	Total CES-D score		.015	.006	.149*	
	Total CES-D score squared		.000	.000	−.101	.001

3	Constant		1.666	.089		
	1992/1993 SASSI-A total score		.220	.020	.296***	
	Total CES-D score		.018	.014	.181	
	Total CES-D score squared		.000	.001	−.176	
	Total CES-D score cubed		.000	.000	.046	.000

*Note:* CES-D=Center for Epidemiological Studies-Depression; STAI=State Trait Anxiety Inventory; BADS=Braver Aggressiveness Dimension Scale; SASSI-A=Substance Abuse Subtle Screening Inventory—Adolescent version; CES-D scale completed during 1992/1993 school year; analyses are reported to three decimal places for clarity.

* *p*<.05.

*** *p*<.001.

Given the large proportion of missing data, we conducted a robustness check of our regression analyses using complete cases (see Supplementary material). These results were almost identical (in terms of Δ*R*^*2*^ values), again finding that baseline levels on the well-being/depression continuum have a near linear relationship with changes in trait anxiety, aggression, and substance misuse over time (regression coefficients are larger than those in Table 2 as multiple imputation estimates relationships more conservatively).

Overall, the regression analyses and graphs provide evidence that the well-being/depression continuum (CES-D total scores) has a near linear relationship with outcome variables over time. Any nonlinearity appears to be of statistical but not practical or clinical significance. These results support our prediction that moving from depression to well-being on the CES-D provides an equal decrease in risk for several other psychological problems, irrespective of position on the well-being/depression continuum.

## 2 Discussion

This study tested two key predictions made by PCP using the example of depressive experiences. We first examined whether the CES-D can be reconceptualised as measuring a continuum that ranges from well-being to depression using a large, heterogeneous sample. These analyses replicated the novel findings of one previous study that used samples of adults and older adults (Wood et al., 2010) and extended them to adolescents. This replication was important because of the potential importance of the suggestion that depression forms a bipolar continuum with well-being. We used a large, ethnically diverse sample that is representative of adolescents residing in Hawaii in order to minimize the likelihood of systematic sampling bias, which could have been introduced had we used a purely community or clinical sample. Taken together with the previous findings (Wood et al., 2010), there is now evidence that the CES-D measures a well-being/depression continuum in adolescents, adults, and older adults. The CES-D total score provides an indication of depressive symptom severity that is based on a combination of the presence/absence of depressive experiences and the presence/absence of well-being. Importantly, this conceptualisation contrasts with other views regarding the inter-relationship between psychological problems and well-being, for instance challenging the Complete State Model of Mental Health's (Keyes, 2003, Keyes, 2007) assertion that “mental illness” and well-being form separate continua.

The theoretical underpinning for our analyses was the suggestion that for a score of zero to occur on the CES-D, a person would have to give all of the negatively worded items (e.g., “I felt sad”) the lowest possible score (“rarely of none of the time”) and all of the positively worded items (e.g., “I enjoyed life”) the highest possible score (“most or all of the time;” (Joseph, 2006, Joseph, 2007, Joseph and Wood, 2010)). Responding in this way would not, therefore, merely indicate an absence of depressive experiences; it would actually indicate the *presence* of well-being (Joseph, 2006, Joseph, 2007, Joseph and Wood, 2010). Conceptualizing depression as one end of a well-being/depression continuum has the advantage of removing arbitrary divides between maladaptive and adaptive. This evidence could therefore be used to make an argument against mental health stigma, since everyone resides somewhere on the well-being/depression continuum. Our conceptualization of depression suggests that individuals who are currently experiencing depressive experiences are not qualitatively different from anyone else; at the moment they are simply further along the well-being/depression continuum towards high depressive experiences.

This is the first study to examine the form of the relationship between the well-being/depression continuum and other psychological problems over time. Our results demonstrate that baseline levels on the well-being/depression continuum have a near linear relationship with outcome variables measured at the same time and one and two years later. It therefore seems desirable to score highly on the well-being pole of the continuum because the higher an individual's well-being, as measured by the CES-D, the less likely that individual is to experience several psychological problems (anxiety symptoms, aggression, problematic substance use) over time. These results point to the value of jointly focusing on increasing well-being and reducing distress, as doing so promotes resilience from developing other psychological problems. They also point to the importance of early intervention in order to prevent people moving away from high levels of well-being. As depression and well-being form a single continuum, fostering high levels of well-being means that individuals have further to move before the onset of depressive experiences; in effect raising the threshold for the onset of depressive experiences. These results join evidence that anxiety can be understood as residing on a calmness-anxiety continuum that also has near linear relationships with other psychological problems over time (Siddaway et al., in press).

Until recent years, the dominant zeitgeist in mental health services has very understandably been to focus on the negative aspects of life and how these can be reduced and alleviated. Our results add to an emerging evidence-base which suggests that we may need to change the way we think about mental health problems. Our results support calls for mental health services to jointly focus on increasing well-being and reducing distress (e.g., The British Psychological Society, 2010) and the World Health Organization's (WHO; 2004) conceptualization of mental and physical health as “a state of complete physical, mental and social well-being and not merely the absence of disease or infirmity.” PCP's explicit call for a balanced and equal focus on the positive and negative aspect of life (Wood & Johnson, in press; Wood and Tarrier, 2010; Wood and Johnson, 2016) aligns well with the view offered by the mental health recovery movement, where “recovery” involves “the establishment of a fulfilling, meaningful life and a positive sense of identity” (Andresen et al., 2003).

People will of course continue to present to mental health services wanting to address their psychological problems. However, the present results could be used to inform an evidence-based discussion regarding when to stop interventions and the advantages and disadvantages of doing so. The evidence presented here suggests that it may be beneficial for clinical interventions to continue beyond the mere absence of depressive symptoms. Although this is clearly less cost-effective in the short-term, it could prove to be the most cost-effective option long-term.

The current results need to be interpreted in light of several limitations, many of which stem from using an archival dataset. Further research is clearly needed to consolidate arguments regarding a more balanced and equally weighted focus on treating problems and increasing well-being. One important limitation of our results arises because the CES-D was not designed to measure a well-being/depression continuum; therefore the well-being items do not measure this pole of the continuum as comprehensively as established scales of well-being. Indeed, the CES-D contains unequal numbers of negatively worded and positively worded items, disproportionately weighting the depression end of the continuum. Developing and validating a scale that specifically measures the well-being/depression continuum is therefore expected to provide additional predictive validity over the CES-D.

It will also be important to establish the form of the relationship between the well-being/depression continuum and psychological problems other than those measured here, as well as measures of positive psychological functioning. Although we used a large, heterogeneous sample, it is possible that the nature and form of the well-being/depression continuum may change over time or vary across particular groups, for instance as a result of cultural changes in how mental health problems are understood. Determining whether and how conceptualisations and inter-relationships of psychological problems and well-being change over time represents a core area of ongoing research. An economic cost value analysis that tests the implications of the well-being/depression continuum conceptualization and when it is most cost effective to stop interventions is particularly needed.

## Role of the funding source

This research is independent research arising from a Medical Research Council-funded Clinical Research Training Fellowship (Grant reference: MR/L017938/1) awarded to Dr Siddaway. The views expressed in this publication are those of the Fellow and not necessarily those of the MRC. The MRC had no role in the writing or submission of this article. Professor Wood was supported by ESRC grant ES/K00588X/1.

## Contributors

AS and AW conceived the study. AS performed the statistical analyses with the guidance of PT. AS wrote the manuscript with the guidance of AW and PT. All authors have approved the final article.

## Footnotes

Appendix A Supplementary data associated with this article can be found in the online version at doi:10.1016/j.jad.2017.02.015.
