# Supplementary material for: The Center for Epidemiologic Studies-Depression (CES-D) scale measures a continuum from well-being to depression: Testing two key predictions of positive clinical psychology
Source: J Affect Disord. 2017 Apr 15;213:180–6. doi: 10.1016/j.jad.2017.02.015 (PMC6191531; doi:10.1016/j.jad.2017.02.015)
Supplement: Supplementary file 1 — Supplementary material [file mmc1.docx]

Supplementary Table I. Results of Regression Analyses Based on Complete Cases Comparing Linear and Nonlinear Effects of CES-D upon Change in Outcome.

| Step | Variables | | *B* | *SE B* | *β* | *ΔR^2^* |
| --- | --- | --- | --- | --- | --- | --- |
|  |  | Trait anxiety as outcome |  |  |  |  |
| 1992/1993 school year (*N* = 3,759) | | |  |  |  |  |
| 1 | Constant | | 10.645 | .199 |  |  |
|  | Total CES-D score | | .725 | .011 | .747*** | .558*** |
| 2 | Constant | | 8.797 | .316 |  |  |
|  | Total CES-D score | | .975 | .035 | 1.005*** |  |
|  | Total CES-D score squared | | -.006 | .001 | -.270*** | .007*** |
| 3 | Constant | | 6.858 | .449 |  |  |
|  | Total CES-D score | | 1.403 | .079 | 1.445*** |  |
|  | Total CES-D score squared | | -.028 | .004 | -1.316*** |  |
|  | Total CES-D score cubed | | .000 | .000 | .643*** | .004*** |
|  |  | Aggression as outcome |  |  |  |  |
| 1992/1993 school year (*N* = 3,910) | | |  |  |  |  |
| 1 | Constant | | 1.862 | .121 |  |  |
|  | Total CES-D score | | .250 | .006 | .527*** | .278*** |
| 2 | Constant | | 1.999 | .194 |  |  |
|  | Total CES-D score | | .232 | .021 | .488*** |  |
|  | Total CES-D score squared | | .000 | .000 | .041 | .000 |
| 3 | Constant | | 1.845 | .277 |  |  |
|  | Total CES-D score | | .266 | .049 | .560*** |  |
|  | Total CES-D score squared | | -.001 | .002 | -.129 |  |
|  | Total CES-D score cubed | | .000 | .000 | .105 | .000 |
| 1993/1994 school year (*N* = 1,935) | | |  |  |  |  |
| 1 | Constant | | 1.274 | .152 |  |  |
|  | 1992/1993 BADS total score | | .564 | .020 | .585*** |  |
|  | Total CES-D score | | .039 | .010 | .085*** | .402*** |
| 2 | Constant | | 1.162 | .236 |  |  |
|  | 1992/1993 BADS total score | | .564 | .020 | .585*** |  |
|  | Total CES-D score | | .054 | .027 | .119* |  |
|  | Total CES-D score squared | | .000 | .001 | -.036 | .000 |
| 3 | Constant | | .819 | .334 |  |  |
|  | 1992/1993 BADS total score | | .564 | .020 | .585*** |  |
|  | Total CES-D score | | .132 | .060 | .288* |  |
|  | Total CES-D score squared | | -.005 | .003 | -.440 |  |
|  | Total CES-D score cubed | | .000 | .000 | .250 | .001 |
| 1994/1995 school year (*N* = 925) | | |  |  |  |  |
| 1 | Constant | | 1.714 | .248 |  |  |
|  | 1992/1993 BADS total score | | .542 | .034 | .521*** |  |
|  | Total CES-D score | | .030 | .016 | .060 | .309*** |
| 2 | Constant | | 1.309 | .382 |  |  |
|  | 1992/1993 BADS total score | | .540 | .034 | .519*** |  |
|  | Total CES-D score | | .088 | .045 | .178* |  |
|  | Total CES-D score squared | | -.001 | .001 | -.124 | .001 |
| 3 | Constant | | .950 | .542 |  |  |
|  | 1992/1993 BADS total score | | .540 | .034 | .518*** |  |
|  | Total CES-D score | | .171 | .099 | .345 |  |
|  | Total CES-D score squared | | -.006 | .005 | -.516 |  |
|  | Total CES-D score cubed | | .000 | .000 | .241 | .001 |
|  |  | Substance misuse as outcome |  |  |  |  |
| 1992/1993 school year (*N* = 3,781) | | |  |  |  |  |
| 1 | Constant | | .223 | .038 |  |  |
|  | Total CES-D score | | .044 | .002 | .334*** | .111*** |
| 2 | Constant | | .221 | .061 |  |  |
|  | Total CES-D score | | .044. | .007 | .336*** |  |
|  | Total CES-D score squared | | -.000 | .000 | -.002 | .000 |
| 3 | Constant | | .250 | .088 |  |  |
|  | Total CES-D score | | .038 | .015 | .288* |  |
|  | Total CES-D score squared | | .000 | .001 | .112 |  |
|  | Total CES-D score cubed | | .000 | .000 | -.070 | .000 |
| 1993/1994 school year (*N* = 1,817) | | |  |  |  |  |
| 1 | Constant | | .228 | .042 |  |  |
|  | 1992/1993 SASSI-A total score | | .608 | .021 | .579*** |  |
|  | Total CES-D score | | .010 | .002 | .080*** | .376*** |
| 2 | Constant | | .236 | .068 |  |  |
|  | 1992/1993 SASSI-A total score | | .608 | .021 | .579*** |  |
|  | Total CES-D score | | .009 | .008 | .071 |  |
|  | Total CES-D score squared | | .000 | .000 | .009 | .000 |
| 3 | Constant | | .200 | .097 |  |  |
|  | 1992/1993 SASSI-A total score | | .608 | .021 | .580*** |  |
|  | Total CES-D score | | .017 | .017 | .138 |  |
|  | Total CES-D score squared | | .000 | .001 | -.150 |  |
|  | Total CES-D score cubed | | .000 | .000 | .098 | .000 |
| 1994/1995 school year (*N* = 866) | | |  |  |  |  |
| 1 | Constant | | .454 | .068 |  |  |
|  | 1992/1993 SASSI-A total score | | .474 | .037 | .414*** |  |
|  | Total CES-D score | | .013 | .004 | .103*** | .211*** |
| 2 | Constant | | .353 | .107 |  |  |
|  | 1992/1993 SASSI-A total score | | .474 | .037 | .414*** |  |
|  | Total CES-D score | | .027 | .012 | .216* |  |
|  | Total CES-D score squared | | .000 | .000 | -.118 | .000 |
| 3 | Constant | | .337 | .154 |  |  |
|  | 1992/1993 SASSI-A total score | | .474 | .037 | .414*** |  |
|  | Total CES-D score | | .031 | .028 | .244 |  |
|  | Total CES-D score squared | | -.001 | .001 | -.186 |  |
|  | Total CES-D score cubed | | .000 | .000 | .042 | .000 |

*Note:* CES-D = Centre for Epidemiological Studies-Depression; STAI = State Trait Anxiety Inventory; BADS = Braver Aggressiveness Dimension Scale; SASSI-A = Substance Abuse Subtle Screening Inventory—Adolescent version; CES-D scale completed during 1992/1993 school year; **p* < .050, ****p* < .001.
